# Intrabiliary ultrasound applied to endoscopic extraction of choledochal stones

**DOI:** 10.2478/abm-2026-0004

**Published:** 2026-04-30

**Authors:** Liang Ye, Huaiyang Cai, Yingwei Wang, Weiqiang Guo

**Affiliations:** Department of Gastroenterology, Liuzhou People's Hospital affiliated to Guangxi Medical University, Liuzhou 545006, China

**Keywords:** choledochal stone, endoscopic retrograde cholangiopancreatography, intrabiliary ultrasound, recurrence

## Abstract

**Background:**

Although ERCP with endoscopic sphincterotomy is effective for choledocholithiasis, small residual stones may be missed by cholangiography, and intraductal ultrasound may improve detection and reduce recurrence.

**Objective:**

To observe the value of intraductal ultrasound (IDUS) in endoscopic extraction of choledocholithiasis.

**Methods:**

We retrospectively analyzed 148 patients with choledocholithiasis who underwent endoscopic retrograde cholangiopancreatography (ERCP) combined with endoscopic sphincterotomy (EST). IDUS was performed to observe whether there were any residual choledocholithiasis stones in those whose previous choledocholithiasis treatment indicated complete clearance; ERCP combined with EST was repeated to retrieve larger residual stones (≥3 mm). Postoperative follow-up was conducted to monitor the recurrence of choledocholithiasis and its influencing factors.

**Results:**

After 148 cases of endoscopic ERCP combined with EST, cholangiography confirmed the complete removal of choledochal stones. Notably, 61 cases were found to have residual stones, and 21 of them had residual stones ≥3 mm; these 21 cases were repeated until the stones were completely removed. After 3–24 months of follow-up, IDUS revealed that the stone recurrence rates were 8.33% (9/108) for 108 cases with complete stone removal and 62.50% (25/40) for 40 cases with residual stones. The difference was statistically significant (*P* < 0.01). The cumulative recurrence rates during the 24-month postoperative period were also significantly different (88.40% and 14.40%, respectively, *P* < 0.01). The results of multivariate analysis showed that choledocholithiasis, common bile duct (CBD) diameter, and angle were independent risk factors for stone recurrence, as indicated by IDUS (*P* < 0.05).

**Conclusions:**

IDUS can detect choledocholithiasis, particularly small stones (<3 mm), which are challenging to visualize with cholangiography. IDUS can assist in ERCP combined with EST to reduce the recurrence of postoperative stones.

Endoscopic retrograde cholangiopancreatography (ERCP) combined with endoscopic sphincterotomy (EST) is the primary endoscopic technique used for treating choledocholithiasis. EST, also known as duodenal papillary sphincterotomy, is the primary endoscopic technique used for treating common bile duct (CBD) stones. It is effective and less invasive [1, 2]. After ERCP combined with EST, some patients are readmitted to the hospital due to the recurrence of stones even after complete removal of choledocholithiasis is confirmed by repeat imaging, with a recurrence rate of approximately 4.0%–24.0% [3, 4]. Undetected residual stones by ERCP may be an important cause of stone recurrence, as contrast may mask small stone fragments [5, 6]. Residual stones of <3 mm are not only difficult to detect but also impossible to remove completely. It has been reported that for patients who undergo an ERCP procedure for stone removal, the duodenal papilla will be incised; therefore, tiny stones measuring <3 mm can usually be self-extracted into the bowel [6]. The results of ERCP are summarized in the following table. Intraductal ultrasound (IDUS) can promptly detect small lesions in the bile ducts. In this study, we investigated the value of IDUS for ERCP combined with EST to reduce the recurrence rate of postoperative choledo-cholithiasis. In cases of stones measuring <3 mm, the duodenal papilla is incised in all patients undergoing ERCP stone retrieval procedures. This results in the self-expulsion of small stones into the bowel.

## Methods

All enrolled patients provided signed informed consent, and this study was approved by the Ethics Committee of Liuzhou People's Hospital affiliated to Guangxi Medical University (Certificate of approval no. 202013). Demographic data of the patients, endoscopic findings, and procedural details were retrieved from hospital information and endoscopic reporting systems, respectively.

### General information

Of note, 148 patients with CBD stones were selected from Liuzhou People's Hospital affiliated to Guangxi Medical University from June 2020 to May 2022. Among them, 52 were male, and 96 were female, aged 19–88 years old, with an average age of (61.0 ± 15.4) years. The maximum diameter of the stones ranged from 5.00 mm to 18.00 mm, with an average of (11.30 ± 3.80) mm. Inclusion criteria: (1) the presence of CBD stones and no gallbladder stones were confirmed by imaging; (2) the stones were extracted by ERCP combined with EST, and the CBD stones were completely removed by ERCP, and the IDUS examination was performed immediately after the operation; and (3) no stone-dissolving medication was given after the removal of the stones. Exclusion criteria: (1) uncorrected coagulation disorders; (2) associated with intrahepatic bile duct stones; (3) malignant tumors; (4) hemodynamic instability; (5) pregnancy; and (6) surgical and other reasons for the change of the anatomical position of the CBD.

### Instruments and methods

An Olympus UM-BS20-26R IDUS instrument was used with an endoluminal probe at 20 Hz. Diazepam (5 mg), pethidine hydrochloride (25–50 mg), and scopolamine butylbromide (20 mg) were injected intravenously before the operation to sedate, provide analgesia, and reduce intestinal spasm. ERCP was performed using a side-view duodenoscope (Olympus TGF-260). This was followed by elective choledochal intubation to confirm the size and number of stones, as well as the measurement of the distal choledochal diameter (the maximum transverse diameter of the choledochal duct) and choledochal angle (the angle between the choledochal jug and the axis of the choledochal duct). Subsequently, duodenal papillary sphincterotomy (EST) was performed. In cases of large choledochal stones, the duodenal papilla was dilated using a disposable dilatation balloon (10–12 mm, 12–15 mm, or 15–18 mm in diameter (Nanwei Medical Technology). Under fluoroscopic and duodenoscopic guidance, stones were extracted using a basket (Olympus) and/or a balloon (Nanwei Medical Technology) until the choledochal stones were completely removed as confirmed by imaging. Later, the CBD was examined by IDUS, and ERCP combined with EST was repeated if relatively large stones (≥3 mm) were suspected.

The efficacy of the treatment was assessed based on the intraoperative IDUS. Complete clearance: No stone remained after stone extraction by ERCP and IDUS, or the residual stone was found to be ≥3 mm by IDUS after stone extraction, and complete clearance was confirmed by repeated stone extraction; presence of residual stone: IDUS after lithotripsy reveals a residual stone <3 mm that cannot be completely removed. Those with completely cleared CBD stones were included in the cleared group, while those with residual stones were included in the residual stone group.

### Procedure

The guidewire is inserted into the CBD through the nipple opening. The subsequent advancement of the balloon along the guidewire is conducted into the duct, CBD and finally to the porta hepatis. The flow of bile is balloon-occluded in order to reduce the backflow of the contrast agent or excessive loss. Subsequent to this, the contrast agent is injected, after which the balloon is gradually dragged. The administration of the contrast agent is followed by the acquisition of an X-ray image, with the purpose of enhancing the clarity of the resultant image.

### Postoperative complications

Post-ERCP pancreatitis (PEP) is persistent abdominal pain that is new or worse than preoperative pain, and lasts for >24 h after an ERCP operation. It is also accompanied by nausea, vomiting, and epigastric tenderness. PEP is diagnosed when serum amylase or lipase is elevated by >3 times the upper limit of the normal value and when the corresponding diagnostic features, such as Computed Tomography (CT)/Magnetic Resonance Imaging (MRI), Operating Room (OR) or abdominal ultrasound, are present. Post-ERCP hyperamylasemia (PEH) is characterized by only transient elevation of amylase after ERCP, in the absence of abdominal pain, hemorrhage, perforation, and cholangitis.

### Postoperative follow-up

After the operation, the patients were followed up once every 3–6 months for a total of 24 months. The patients with suspected recurrence of choledocholithiasis underwent imaging and laboratory examinations. ERCP was repeated to confirm the diagnosis when abnormalities were detected. Subsequently, the patients were divided into recurrence and non-recurrence groups.

### Statistical analysis

The data were statistically analyzed using SPSS 24.0. Measurement information was expressed as ± s and compared using Student's *t*-test. The χ^2^ test or Fisher's exact probability method was used to compare the count data. Variables with statistically significant differences in univariate analysis were included in the multivariate logistic regression model. The Kaplan–Meier method was used for survival analysis to observe the relationship between complete removal of choledochal struvite and stone recurrence. A *P*-value of <0.05 was considered to indicate a statistically significant difference.

## Results

**Figure 1.**
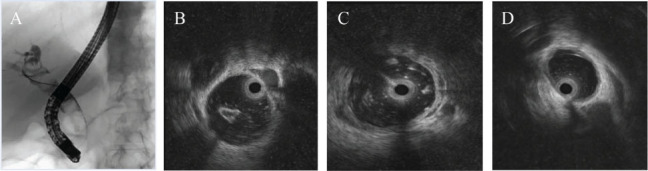
Female patient, 61 years old, with choledocholithiasis, who underwent ERCP combined with EST for stone extraction. **(A)** ERCP showed multiple stone shadows with diameters of 10–14 mm in the whole section of the choledocholithiasis (arrows). **(B, C)** ERCP combined with EST, no stone was seen on imaging after stone removal, and IDUS showed 2 <3 mm diameter stones in the middle part of the CBD (arrows). **(D)** Repeat extraction after lithotripsy, IDUS shows that the lower part of the CBD is ≥3 mm in diameter, and the residual stone has been removed. CBD, common bile duct; ERCP, endoscopic retrograde cholangiopancreatography; EST, endoscopic sphincterotomy; IDUS, intraductal ultrasound.

In 148 cases, complete removal of choledocholithiasis was confirmed by cholangiography after ERCP combined with EST. The IDUS revealed that out of 148 cases, 87 had no remaining stones. Among the remaining cases, 61 (41.22%) had residual choledocholithiasis. Of these, 21 cases with residual stones ≥3 mm were successfully removed after repeated stone removal (**Figure 1**), while 40 cases had residual choledocholithiasis of <3 mm. In total, 108 cases were completely cleared (clearance group), while 40 cases still had residual stones (residual stone group) after the stone removal. After the extraction of stones, 108 cases were completely removed (removal group), while 40 cases had residual stones (residual stone group). No serious adverse reactions were observed during or after the operation.

### Factors affecting the residual stones in the CBD

There were no statistically significant differences in gender, age, and maximum stone diameter between the removal group and the residual stone group (*P* < 0.05). The diameter of the CBD was smaller than that of the residual stone group (*P* < 0.01), and the angle of the CBD was larger than that of the residual stone group (*P* < 0.01), as shown in **Table 1**.

**Table 1.** Comparison of data related to patients with complete stone removal and residual stones after ERCP combined with EST stone extraction

**Group**	**Male/female**	**Age (years)**	**Diameter of CBD (mm)**	**Angle of CBD (°)**	**Maximum diameter of stones (mm)**
Removal group (n = 108)	36/72	60.10 ± 15.30	10.20 ± 2.58	143.76 ± 10.65	10.03 ± 3.91
Residual stone group (n = 40)	16/24	62.30 ± 18.70	12.67 ± 3.21	130.98 ± 11.41	12.15 ± 3.13
χ^2^/*t* value	0.98	0.72	5.17	6.91	1.02
*P* value	0.32	0.46	<0.01	<0.01	0.32

CBD, common bile duct; ERCP, endoscopic retrograde cholangiopancreatography; EST, endoscopic sphincterotomy.

### Incidence of complications

Postoperative hyperamylasemia was higher in the residual stone group than in the removal group (9.3% vs. 20.0%), and this difference was statistically significant (*P* < 0.05). The incidence of postoperative pancreatitis was lower in the removal group than in the residual stone group (18.7% vs. 20.5%). Complete stone removal helps patients to recover more quickly; however, the difference in postoperative pancreatitis between the 2 groups was not statistically significant (*P* > 0.05). Hemorrhage, perforation, or cholangitis were not experienced by either group of patients.

### Factors affecting stone recurrence

During the follow-up period, 9 cases in the removal group and 25 cases in the residual stone group had stone recurrence, and the recurrence rates were 8.33% (9/108) and 62.50% (25/40), respectively, with statistically significant differences (χ^2^ = 43.24, *P* < 0. 01). The time of recurrence ranged from 14 months to 22 months after surgery, with a median time of 18 months. There were no statistically significant differences between the recurrence and non-recurrence groups in terms of gender, age, and maximum stone diameter (*P* < 0.05). In the recurrent group, the diameter of the CBD was larger than that of the non-recurrent group (*P* < 0.05), and the angle of the CBD was smaller than that of the non-recurrent group (*P* < 0.05), as shown in **Table 2**. The results of multivariate analysis revealed that the residual choledochotomy stone, the diameter of the CBD, and the angle of the CBD observed in the IDUS were identified as independent risk factors for stone recurrence (*P* < 0.05), as depicted in **Table 3**.

**Table 2.** Comparison of relevant data of patients with and without recurrence after ERCP combined with EST stone extraction

**Group**	**Male/female**	**Age (years)**	**Diameter of CBD (mm)**	**Angle of CBD (°)**	**Maximum diameter of stones (mm)**
Recurrence group (n = 34)	11/23	66.60 ± 15.80	12.63 ± 3.46	131.79 ± 12.05	11.40 ± 0.38
Non-recurrence group (n = 114)	41/73	59.20 ± 16.10	10.79 ± 2.88	140.49 ± 12.13	11.30 ± 0.39
χ^2^/*t* value	0.97	2.21	3.12	3.67	0.09
*P* value	0.32	0.28	<0.01	<0.01	0.94

CBD, common bile duct; ERCP, endoscopic retrograde cholangiopancreatography; EST, endoscopic sphincterotomy.

**Table 3.** Recurrence of choledocholithiasis after ERCP combined with EST lithotripsy risk factor analysis

**Factor**	***P***	**OR**	**Wals**	**β**
Remnant biliary stone	<0.05	12.72	35.39	2.54
Diameter of CBD	<0.05	1.24	12.50	0.22
Angle of CBD	<0.05	0.95	11.50	11.50

CBD, common bile duct; ERCP, endoscopic retrograde cholangiopancreatography; EST, endoscopic sphincterotomy.

**Figure 2.**
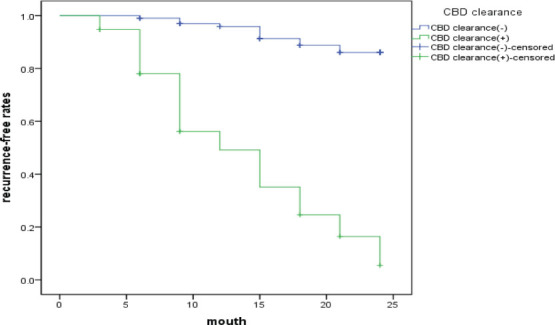
Kaplan–Meier curve of the cumulative no recurrence rate in the cleared versus residual stone group after ERCP combined with EST choledochotomy. CBD, common bile duct; ERCP, endoscopic retrograde cholangiopancreatography; EST, endoscopic sphincterotomy.

At 24 months after surgery, the cumulative recurrence-free rate of choledocholithiasis in the clearance group was 88.40%, while that in the residual stone group was only 14.40% (χ^2^ = 39.46, *P* < 0.01), as shown in **Figure 2**.

## Discussion

Recurrent choledocholithiasis stones after ERCP combined with EST extraction may primarily result from residual stones in the CBD rather than new choledocholithiasis or migration of gallbladder stones. Previous studies [7, 8] reported that the recurrence of choledocholithiasis after ERCP combined with EST is often attributed to incomplete intraoperative removal of small choledocholithiasis, particularly those <3 mm in size. Recurrence tends to be concentrated in the first 3 years following the procedure, which aligns with the findings of the current study. X-ray cholangiography was not effective in detecting small choledochal stones, typically measuring <3 mm [9]. Thus, small stones may represent an early stage of choledochal stone formation [10].

Fluoroscopic cholangiographic images are currently the primary method of determining successful CBD stone removal [11]. However, ERCP produces a large number of small stone fragments, which may lead to recurrent stone formation [12]. Residual stones are found in approximately one-third of cases when observed using cholangioscopy after ERCP stone removal [5]. Cholangioscopy enables direct visualization of the location, morphology, and size of residual stones, as well as of the bile ducts for bleeding and injury. Many studies have demonstrated the high sensitivity of choledochoscopy in detecting residual CBD stones, with standard cholangiography missing residual stones ranging from 25.3% to 34% [13]. However, cholangioscopy is a single-use procedure with a high cost per visit and is only available at a few large medical centers. In contrast, IDUS is reusable, has a low cost per visit (about one-twentieth of the cost of cholangioscopy), and is less costly to perform. Therefore, IDUS is better suited to routine screening.

IDUS is an auxiliary diagnostic method with high sensitivity and specificity. It has the advantages of being minimally invasive, low-cost, and reproducible [11]. In this study, despite cholangiography after ERCP combined with EST showing complete stone removal, IDUS still detected 41.22% (61/148) of choledocholithiasis remnants. Among these remnants, 14.19% (21/148) were ≥3 mm in size, necessitating a repeat extraction to remove the residual stone. The results of multivariate analysis showed that the risk of recurrence was 12 times higher in the group with stone remnants than in the group with stones removed (OR = 12.72). This indicates that effective detection of choledocholithic remnants and complete removal of as many remnants as possible are necessary to minimize postoperative recurrence. For choledocholithiasis that is difficult to detect accurately by cholangiography, especially stones smaller than 3 mm, IDUS can be used as a complementary tool to effectively detect small stones. This can help increase the detection and removal rate of choledocholithiasis and further reduce the recurrence rate [8]. The relationship between the choledochal angle and choledocholithiasis has not yet been clarified [6]. Studies have confirmed that the angle of the CBD is an independent factor influencing the recurrence of choledocholithiasis [12]. The localized biliary stasis resulting from the narrow angle of the CBD may be a significant factor in the development of choledocholithiasis. Additionally, the acute angle of the distal part of the CBD is a primary factor contributing to the challenge of extracting choledocholithiasis. The results of this study indicate that the angle of the CBD is associated with the presence of stone remnants in the CBD. A smaller angle impacts the procedure for stone removal, consequently raising the likelihood of recurrence.

The diameter of the CBD is a risk factor for recurrent choledocholithiasis. A large diameter of the CBD may lead to the recurrence of stones because of incomplete removal by the mesh basket or balloon [7]. A larger CBD diameter increases the likelihood of recurrent choledocholithiasis, possibly due to biliary stasis in the bile ducts or biliary reflux of duodenal fluid [5]. Cholangiography is less sensitive to small stones, and larger diameters of the CBD tend to retain smaller stones. In this study, the diameter of the choledochotomy was significantly larger in the group with residual stones than in the group with cleared stones. This suggests that a larger choledochotomy diameter is associated with a higher risk of stone recurrence.

In conclusion, IDUS can detect residual stones in the CBD after ERCP and EST. It can assist ERCP and EST in reducing the retention and recurrence of CBD stones during ERCP and EST procedures. However, the sample size of this study is small, and it is a single-center analysis. Further observation is needed in a large-sample, multicenter study.
